# Influencing factors on the time to CT in suspected pulmonary embolism: an explorative investigation

**DOI:** 10.1038/s41598-024-59428-2

**Published:** 2024-04-16

**Authors:** Daniel Koehler, Ann-Kathrin Ozga, Isabel Molwitz, Farzad Shenas, Sarah Keller, Gerhard Adam, Jin Yamamura

**Affiliations:** 1 Department of Diagnostic and Interventional Radiology and Nuclear Medicine, University Medical Center Hamburg-Eppendorf, Martinistraße 52, 20246 Hamburg, Germany; 2 Institute of Medical Biometry and Epidemiology, University Medical Center Hamburg-Eppendorf, Martinistraße 52, 20246 Hamburg, Germany; 3 Department of Radiology, Charité - Universitätsmedizin Berlin, Corporate Member of Freie Universität Berlin, Humboldt-Universität zu Berlin, and Berlin Institute of Health, Charitéplatz 1, 10117 Berlin, Germany

**Keywords:** Respiratory tract diseases, Health care economics

## Abstract

Pulmonary embolism is a potentially fatal condition with increased mortality if anticoagulation is delayed. This study aimed to find influencing factors on the duration from requesting a computed tomography (CT) pulmonary angiography (CTPA) to performing a CTPA in suspected acute pulmonary embolism. In 1849 cases, automatically generated time data were extracted from the radiological information system. The impact of the distance to the scanner, case-related features (sector of patient care, triage), and workload (demand for CTs, performed CTs, available staff, hospital occupancy) were investigated retrospectively using multiple regression. The time to CTPA was shorter in cases from the emergency room (ER) than in inpatients and outpatients at distances below 160 m and 240 m, respectively. While requests from the ER were also performed faster than cases from regular wards (< 180 m), no difference was found between the ER and intensive care units. Compared to “not urgent” cases, the workflow was shorter in “urgent” (− 17%) and “life-threatening” (− 67%) situations. The process was prolonged with increasing demand (+ 5%/10 CTs). The presented analysis identified relevant in-hospital influences on the CTPA workflow, including the distance to the CT together with the sector of patient care, the case triage, and the demand for imaging.

## Introduction

Pulmonary embolism (PE) is a frequent diagnosis in emergency medicine with a potentially fatal course of disease^1–3^. Hemodynamically unstable patients and individuals with a high clinical probability of PE should undergo computed tomography pulmonary angiography (CTPA) as the standard imaging method without further diagnostic tests. Otherwise, D-dimer testing is recommended^3,4^. D-dimer forms when cross-linked fibrin is degraded, leading to elevated plasma levels in most cases of venous thromboembolism^5^. If D-dimer levels are elevated in patients with a low or intermediate risk of PE, CTPA is recommended^3,4^.

PE-related mortality rates decreased in the past decades^6,7^. One potential reason for this development may have been the progress in imaging techniques like CTPA^8^, which has excellent sensitivity and specificity (94% and 98%, respectively)^9^. Since its introduction, multiple studies have described an increase in the usage of CTPA accompanied by a decrease in diagnostic yield trends^10–12^. Subgroups that were reported to be especially at risk of overutilization of CTPA were young patients and females^13^, in whom the associated increased radiation exposure is particularly critical. The topic becomes even more complex considering that the decline in mortality rates slowed or demonstrated a rebound increase among young and middle-aged adults^7^. PE remains a relevant cause of death, especially in women between 20 and 50 years of age (up to 16 deaths per 1000 deaths)^6,7^. Early anticoagulation was reported to reduce mortality in acute PE (in-hospital mortality 1.4% vs. 6.7% and 30-day mortality 4.4% vs. 15.3%, respectively)^14,15^. This implies that the duration from disease onset to treatment should be as short as possible. The same theorem has long been established in emergencies like ischemic stroke^16–18^ or myocardial infarction^19–21^. In these conditions, guidelines emphasize the need for rapid diagnostic tests and timely initiation of treatment^22–25^. A similar set of recommendations may also improve outcomes in patients with PE. Differences in the temporal distribution and quality of patient care have already been described in multiple studies^26–29^. A recent meta-analysis regarding the diagnostic delay of PE focused on patient factors like symptoms and comorbidities^30^. The subsequent step involves elucidating potential factors that may impact the in-hospital diagnostic process for suspected PE. Therefore, this explorative study aimed to investigate if factors like the distance to the CT scanner, case-related features, and workload influence the duration of the CTPA workflow in patients with suspected acute PE.

## Materials and methods

### Study cohort

The local ethics committee of the Ärztekammer Hamburg (Germany) approved this retrospective study and waived the requirement for informed consent (No. PV7353). All methods were conducted in compliance with the latest Declaration of Helsinki. Data were collected at a tertiary referral hospital comprised of > 30 departments that provide care for approximately 500,000 patients per year. The investigated study population represents a subgroup of a previously published cohort admitted for CTPA in suspected PE in 2013 and 2018^29^. In brief, patients from all departments were included in the analysis if the clinical suspicion of PE was explicitly stated in the written request for CT. CTPA due to other indications, ambiguous requests, or incidental PE findings were excluded. Additionally, all electronical patient files were reviewed to differentiate cases with suspected chronic PE from acute PE, only including acute cases in the current analysis (n = 1849).

### Acquisition of time data

The duration of the CTPA workflow was defined as the time from the CTPA request (ToR) to the time a study was performed (ToS). Every patient-related documentation in the radiological information system (Centricity, GE Healthcare, Chicago, IL, USA) has an automatically generated timestamp. The ToR, corresponding to the time the requester finalized the request, and the ToS, defined by the tag 0008,0030 of the standard of Digital Imaging and Communications in Medicine as the time a study starts^31^, were used in the analysis.

### Influencing factors

The selection of potential influencing factors on the ToR to ToS was hypothesis-driven and based on clinical experience and reports on other emergencies^32–34^. The included factors were subdivided into three categories: distance to the CT scanner, case-related influences, and workload measures. Data were extracted from the hospital information system (Soarian Clinicals, Cerner Corp., Kansas City, MO, USA), the radiological information system, or measured.

### Distance to the CT scanner

The distance between the requesting department and the respective CT scanner was measured using a measuring wheel to investigate the influence of the different departments’ locations on the CTPA workflow.

### Case-related features

Cases were assigned to three different sectors of patient care (i.e., emergency room [ER], inpatient, outpatient) based on the requesting department as specified in the digital request form in the radiological information system. This parameter was chosen to investigate the potential influence of different clinical settings, organizational structures, and training in emergency medicine on the ToR to ToS. The category of “inpatient” was further subdivided into requests from intensive care units (ICU) and regular wards to allow separate analyses of departments specialized in emergency care (ER, ICU) and regular care (regular ward, outpatient).

Next, the case triage divided into “not urgent”, “urgent”, and “life-threatening” was seen as a possible influence on the workflow. The treating physicians routinely assessed the priority based on the anamnesis, physical examination, and transthoracic echocardiography if clinically indicated.

### Workload

Workload-related influencing factors were pooled in three time intervals (I1: 6 am–2 pm, I2: 2 pm–10 pm, I3: 10 pm–6 am). This included the median number of requested and performed CT scans during the interval of the ToR as a measure of demand. Conversely, the number of staff on the CT team was integrated into the model to analyze the influence of available resources from the radiology department on the ToR to ToS. The number of available CT scanners correlated highly with the number of staff and was, therefore, omitted.

Lastly, the occupancy rate of hospital beds on the day of the ToR was included as a surrogate parameter for the overall workload in the hospital, indirectly influencing the CTPA workflow.

### Statistical analyses

Patient characteristics were summarized with descriptive statistical methods. Absolute and relative frequencies were given for categorical data. Continuous variables were described using median and interquartile range (IQR). A linear relationship between the influencing factors (i.e., independent variables) and the duration of the CTPA workflow (i.e., dependent variable) was hypothesized. Two linear regression models were fitted. In the first, patients from the ER, inpatients, and outpatients were included. In the second, the sectors “ER” and “outpatient” remained unchanged, while the sector of “inpatient” was replaced by its subgroups “ICU” and “regular ward”. Interaction terms between the distance to the CT scanner and the sectors of patient care were included in both models to account for the structural design of the investigated center with one CT scanner close to the ER and two scanners in a central position of the clinic. To best reflect the concept of multiple factors influencing the duration of the CTPA workflow, all selected independent variables were included in the models. The ToR to ToS was transformed using the natural logarithm to adjust for the right-skewed distribution of residuals. The resulting coefficients (β) and the corresponding 95% confidence intervals (CI) were transformed back by exponentiation to the base of *e* (exp^β^). Average marginal effects of the distance to the CT scanner and the sector of patient care were estimated, with all other independent variables kept as is. All reported *P*-values were descriptive and were not adjusted for multiple testing due to the explorative design of the study. Missing values were not imputed.

STATA® version 17.0 (STATA Corp, College Station, Texas, USA) was used to conduct all statistical analyses and produce all figures.

## Results

Of 1849 cases, 865 (46.8%) patients were female and 984 (53.2%) patients were male (median age 66, IQR 53–75). CTPA were positive in 338 (18.3%) cases (Supplementary Table S1). The median ToR to ToS was 66 min (IQR 29–138).

### Influencing factors

**Figure 1:**
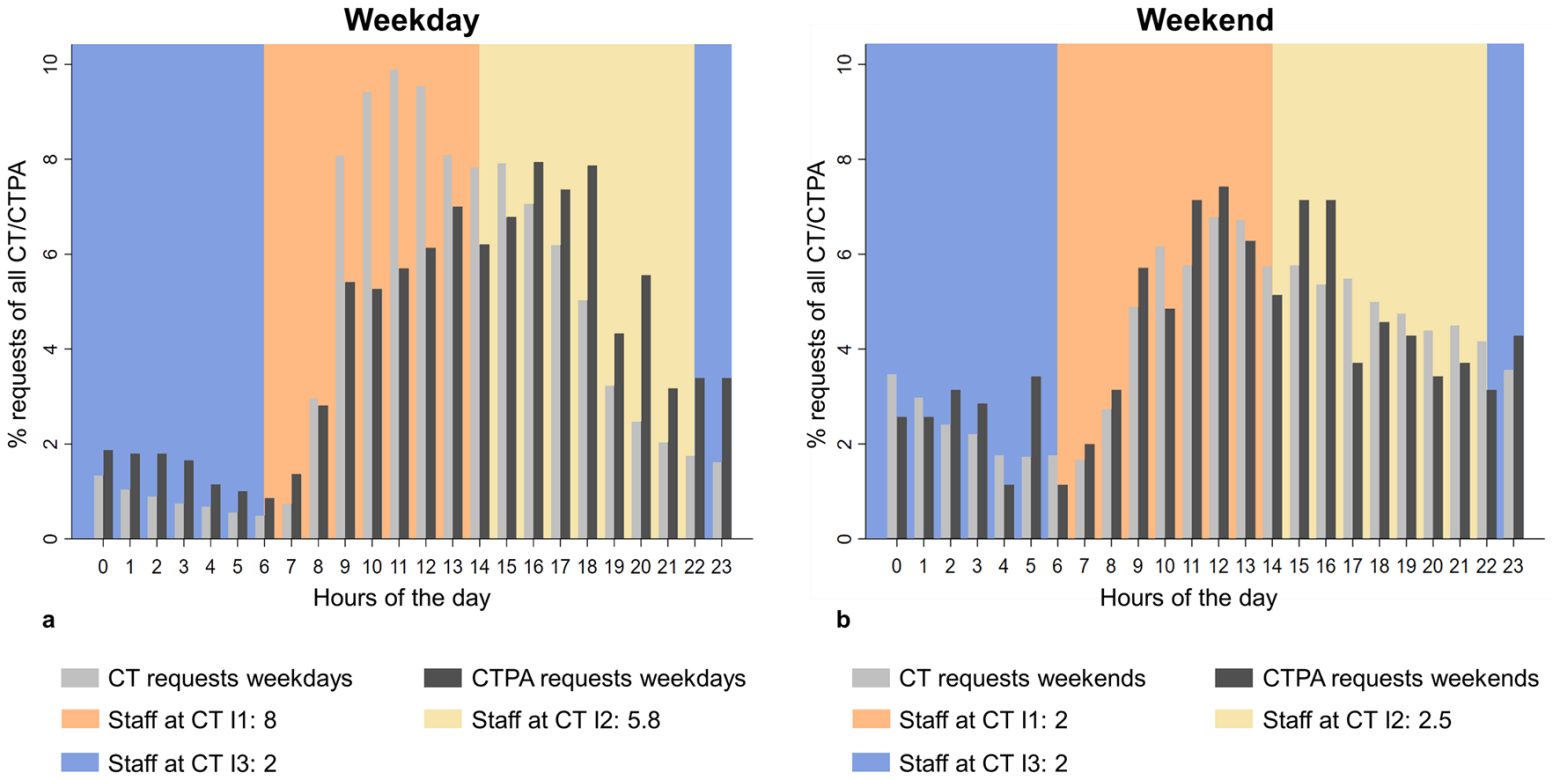
Relative demand for CT/CTPA per hour of the day during the study period and median number of staff available during intervals I1 (6 am–2 pm), I2 (2–10 pm), and I3 (10 pm–6 am) on weekdays (**a**) and weekends (**b**).

Details regarding the independent variables were summarized in Table 1. The distance between the requesting units and the CT locations ranged from 25 to 647 m (Table 1, Supplementary Table S2). Distances between departments and the respective CT scanners were shortest for patients from the ER (ER: median 25 m, IQR 25–25; inpatient: median 227, IQR 98–249; outpatient: median 220 m, IQR 127–221). Most CTPA were requested for patients from the ER (49.8%) or inpatients (47.6%). Only 2.6% of cases with suspected acute PE came from outpatient clinics. The distribution of priority groups differed between the three sectors of patient care. However, most requests were seen as urgent in all three sectors (Supplementary Table S3). Demand for CT imaging and CTPA differed throughout the 24 h of the day, demonstrating peaks of demand during the daytime (Fig. 1).

**Table 1:** Descriptive statistics of independent variables.

Variable	Value
Distance to CT (meters)	65 (IQR 25–227)
Sector	
ER	921 (49.8%)
Inpatients	880 (47.6%)
Outpatients	48 (2.6%)
Priority	
Not urgent	501 (27.1%)
Urgent	981 (53.1%)
Life-threatening	367 (19.9%)
CT requests at ToR	48 (IQR 30–60)
CTs performed at ToR	44 (IQR 31–64)
CT staff at ToR	5.8 (2.5–8)
Occupancy at ToR	89% (IQR 85%–92%)

Categorical variables are given in absolute and relative frequencies. Continuous variables are described using median and interquartile range.

*IQR* interquartile range, *ToR* time of request.

### Multiple linear regression models

**Figure 2:**
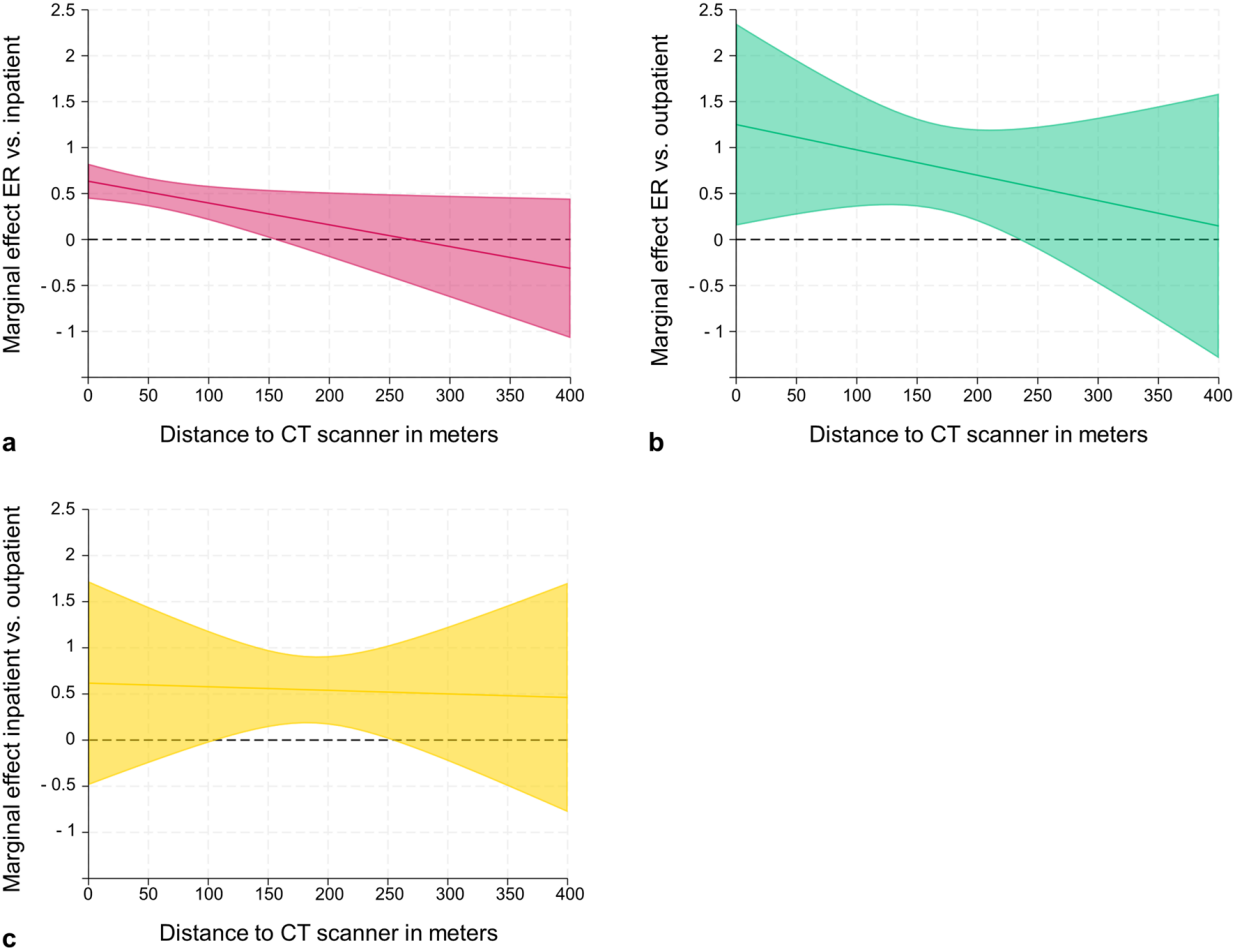
Average marginal effects of the sector of patient care (emergency room [ER] versus inpatient, (**a)** ER versus outpatient, (**b)** inpatient versus outpatient, (**c**) in relation to the distance from the requesting department to the CT scanner. The shaded areas represent the respective 95% confidence intervals.

The ToR to ToS increased with the distance between the ER and the CT scanner (+ 33% per 100 m) (Table 2). The derived average marginal effects showed no influence of the distance on the ToR to ToS in the other sectors of patient care (Table 3). If the distance to the CT scanner was 0 m, the duration of the workflow would have been longer in inpatients (ER vs. inpatient: 1.89, 95% CI 1.56–2.28, *P* < 0.001), and in outpatients (ER vs. outpatient: 3.49, 95% CI 1.16–10.49, *P* = 0.026) compared to the ER. However, Fig. 2 illustrates the influence of the distance together with the sector of patient care on the duration of the CTPA workflow more adequately. The average marginal effects of the ER decreased with increasing distance to the CT scanner. The process was shorter for requests from the ER than in inpatients and outpatients at distances below 160 m and 240 m, respectively. The ToR to ToS was also shorter in cases from the ER compared to regular wards (< 180 m) and outpatient clinics (< 240 m) in the second model (Supplementary Tables S4 and S5, Supplementary Fig. S1). No difference was found between the ER and ICUs. Similar to the ER, the average marginal effects of the ICU compared to regular wards and outpatient clinics decreased with increasing distance (Supplementary Fig. S1).

**Table 2:** Multiple linear regression of ToR to ToS.

	exp^β^	95% CI	*P*-value
Distance (per 100 m)	1.33	1.08–1.62	0.006
Sector of patient care			
ER versus inpatient	1.89	1.56–2.28	< 0.001
ER versus outpatient	3.49	1.16–10.49	0.026
Inpatient versus outpatient	1.85	0.61–5.59	0.274
Priority			
Not urgent versus urgent	0.83	0.73–0.95	0.005
Not urgent versus life-threatening	0.33	0.28–0.39	< 0.001
Urgent versus life-threatening	0.4	0.34–0.46	< 0.001
CT requests at ToR (per 10 CTs)	1.05	1–1.1	0.037
CTs performed at ToR (per 10 CTs)	1.03	1–1.07	0.069
CT staff at ToR	1.12	1.08–1.15	< 0.001
Occupancy at ToR	0.67	0.25–1.8	0.426
Interaction Sector of patient care & distance to CT			
ER (Reference)	1.33	1.08–1.62	0.006
Inpatient	0.79	0.64–0.98	0.03
Outpatient	0.76	0.42–1.37	0.359

*CI* confidence interval; *ER* emergency room; *exp*^*β*^ exponentiated regression coefficient β to the base of *e*; *ICU* intensive care unit; *reg.* regular; *ToR* time of request.

**Table 3:** Average marginal effects of the distance from the requesting department to the CT scanner (per 100 m) on the ToR to ToS for each sector of patient care.

	exp^β^	95% CI	*P*-value
Sector of patient care			
ER	1.33	1.08–1.62	0.006
Inpatient/reg. ward	1.05	0.98–1.12	0.183
Outpatient	1.01	0.58–1.75	0.981

*CI* confidence interval; *ER* emergency room; *exp*^*β*^ exponentiated regression coefficient β to the base of *e*; *ICU* intensive care unit; *reg.* regular.

Next, the clinically assessed case triage had a substantial influence on the ToR to ToS, reducing the time needed to perform a CTPA by 17% in “urgent” cases and by 67% in “life-threatening” situations compared to the “not urgent” category. The workflow was prolonged with rising demand for CT imaging (5% per 10 CTs). Lastly, the ToR to ToS had a positive association with the number of available staff on the CT team (12% per member of staff). However, there seemed to be a parallel increase between the demand for CT and CTPA that correlated with the number of staff on duty (Fig. 1).

## Discussion

The presented analysis of automatically generated time data and healthcare metadata revealed that the distance to the CT scanner together with the sector of patient care, the clinical case triage, and the overall demand for CTs were relevant influencing factors on the duration of the CTPA workflow in patients with suspected acute pulmonary embolism.

Radiology departments play a central role in the care of patients with suspected PE due to the importance of imaging tests like CTPA^3,4^. The current body of literature regarding the time to diagnosis in PE primarily addressed individual risk factors for delays rather than systemic in-hospital reasons^30^. However, these analyses were established in other conditions, such as stroke. Here, complex pre- and in-hospital factors that influenced the time to diagnosis were identified^32^. For example, Ferrari et al. described a delay in stroke imaging if the radiology department was more than two minutes away from the stroke unit or emergency department^34^. In the analyzed cohort, the influence of the distance to the CT scanner was particularly relevant in cases from the ER (+ 33% per 100 m). Furthermore, differences in the duration of the CTPA workflow between the sectors of patient care were distance dependent (Fig. 2 and Supplementary Fig. S1). Above 160 m, the reducing effect of the ER compared to inpatients/regular wards and outpatients diminished. Similarly, the average effect of ICUs compared to these sectors decreased with increasing distance to the scanner. Possible explanations for these observations could be the advanced emergency training of staff working in the ER or in intensive care and higher ratios of staff to patients. This allows for the faster preparation of patients and the more prompt organization of transportation than in wards with lower staffing levels. Furthermore, patients can be transported by medical staff over short distances if enough personnel are available. For longer distances, it is usually necessary to deploy dedicated transportation services, slowing down the process in all sectors. Further studies are needed to investigate if these findings can be reproduced at other sites and in different clinical settings. Evidence based hospital layouts could improve patient flows and enhance interdisciplinary collaboration, such as in PE response teams^3^. These teams aim to evaluate cases in real-time and support clinical decision-making. A recent study demonstrated that they can reduce the time to diagnosis in patients with PE^35^. While no specialized response team was implemented at the investigated institution, the already integrated triage system proved to have the desired influence on the time needed to perform a CTPA. Imaging was done 17% faster in “urgent” cases and 67% faster in “life-threatening” situations compared to the “not urgent” category.

Demand for CT imaging had the opposite effect on the duration of the CTPA process, increasing the ToR to ToS by 5% for every ten additionally requested CT scans. Similarly, Reznek et al. reported a significant association between emergency department crowding and delays of CT scans in stroke patients^33^. In contrast, an unexpected positive correlation between the number of staff on the CT team and the duration of the CTPA workflow was found (12% per staff member). As illustrated in Fig. 1, the demand for CT and CTPA demonstrated a substantial overlap with the number of staff on the CT team, suggesting that the number of requests represented a confounding factor. This implies that more resources are needed to meet the CT demand during these periods.

The presented results indicate the potential value of analyzing automatically generated time data and healthcare metadata in quality management (QM) and business intelligence (BI). By integrating multivariable models into QM and BI tools, new structural layouts, future investments into equipment, and changes to staffing rosters may be simulated before they are implemented, helping to scale a radiology department appropriately. Moreover, the same programs would allow real-time monitoring of workflows, enabling physicians to evaluate adjustments without delay. Most radiology departments are already well prepared for similar analyses due to their high standard of digitalization. Unlike manually entered values, automatically generated data (e.g., timestamps) are highly reliable and do not require additional efforts to be created. By optimizing diagnostic workflows, patient care will improve, and departments can operate more cost-effectively.

Some limitations need to be acknowledged in this retrospective analysis. To avoid the inclusion of falsely labeled cases, only requests that explicitly stated the suspicion of PE were included, potentially causing a selection bias. Second, the analyzed data originated from a single institution, and influencing factors may vary between facilities (e.g., hospital layout, sectors of patient care, etc.). At the investigated center, the subgroup of patients admitted from outpatient clinics was comparatively small so that no reliable conclusions could be drawn. However, the sector of patient care was one of the relevant influencing factors on the duration of the CTPA workflow. It should be analyzed in more depth and in larger cohorts to identify which features led to the observed differences (e.g., organizational structures, staff training, etc.). Similarly, the case triage, which successfully influenced the time to CTPA, should be investigated further to analyze how the existing system influenced workflows and if it can be improved to enhance patient care. Lastly, interactions between distinct processes in a hospital are extremely complex. To fully comprehend their influence on one another, analyses of each procedure are needed, exceeding the study's scope.

## Conclusion

In-hospital factors like the distance to the CT scanner together with the sector of patient care, the clinical case triage, and the overall demand for CTs were identified as relevant influences on the duration of the CTPA workflow. Further investigations are necessary to validate these findings at other centers and in different clinical settings. They could become important parameters in quality management of emergency workflows.

### Supplementary Information

Supplementary Information 1.

Supplementary Information 2.

## Data Availability

The datasets generated during and/or analysed during the current study are available from the corresponding author on reasonable request.

## Abbreviations

BI: Business intelligence
CI: Confidence interval
CTPA: Computed tomography pulmonary angiography
ER: Emergency room
ICU: Intensive care unit
IQR: Interquartile range
PE: Pulmonary embolism
QM: Quality management
ToR: Time of request
ToS: Time of study
