# Supplementary figures and images for: Influencing factors on the time to CT in suspected pulmonary embolism: an explorative investigation
Source: Sci Rep. 2024 Apr 16;14:8741. doi: 10.1038/s41598-024-59428-2 (PMC11021441; doi:10.1038/s41598-024-59428-2)

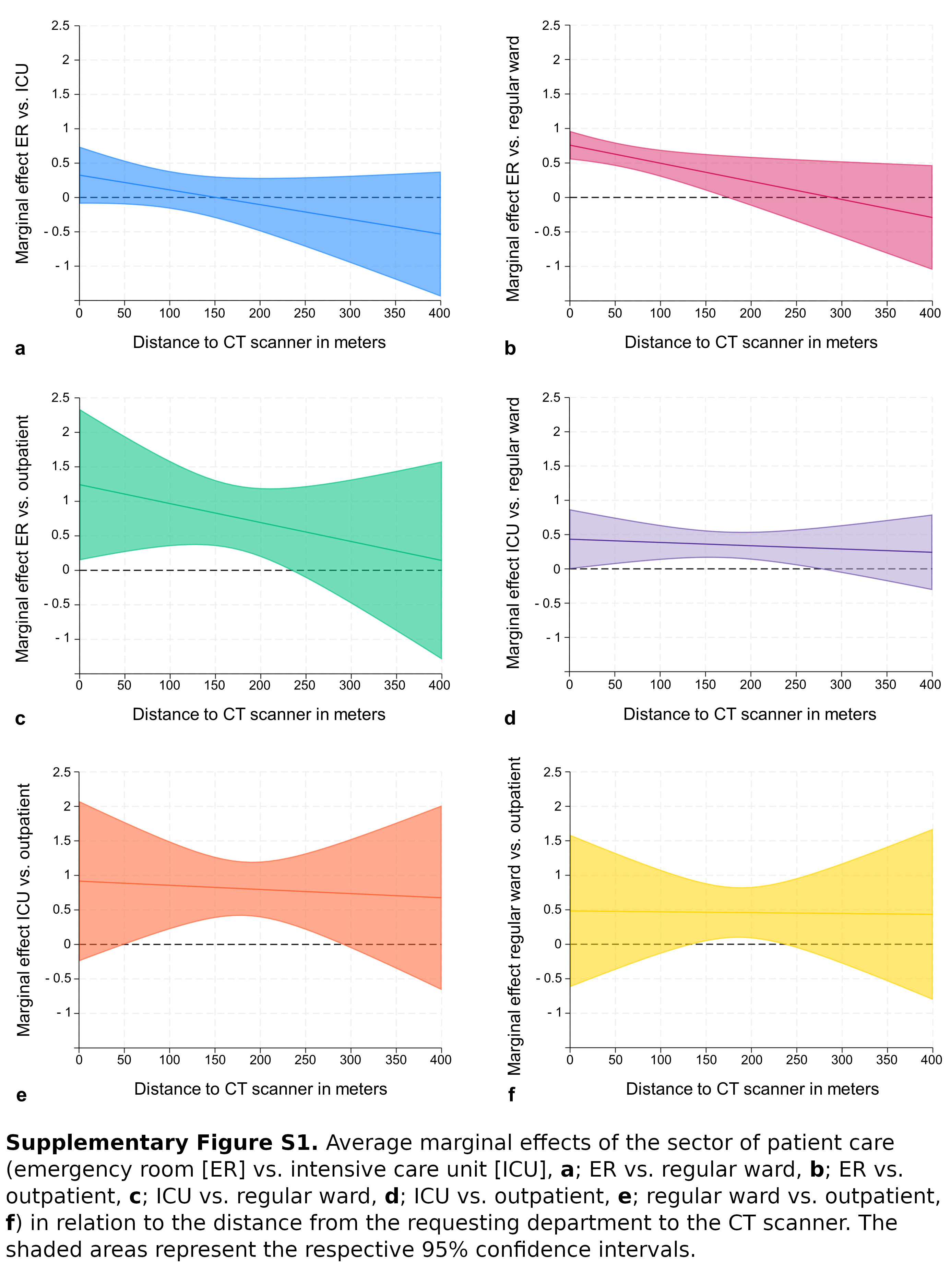

Supplement: Supplementary file 1 — Supplementary Information 1. [file 41598_2024_59428_MOESM1_ESM.tif]
