# Supplementary material for: Influencing factors on the time to CT in suspected pulmonary embolism: an explorative investigation
Source: Sci Rep. 2024 Apr 16;14:8741. doi: 10.1038/s41598-024-59428-2 (PMC11021441; doi:10.1038/s41598-024-59428-2)
Supplement: Supplementary file 2 — Supplementary Information 2. [file 41598_2024_59428_MOESM2_ESM.pdf]

Influencing factors on the time to CT in suspected pulmonary embolism: An explorative investigation

Daniel Koehler[1], Ann-Kathrin Ozga[2], Isabel Molwitz[1], Farzad Shenas[1], Sarah Keller[3], Gerhard Adam[1], Jin Yamamura[1]

Affiliations

[1] Department of Diagnostic and Interventional Radiology and Nuclear Medicine, University Medical Center Hamburg-Eppendorf, Martinistraße 52, 20246 Hamburg, Germany

[2] Institute of Medical Biometry and Epidemiology, University Medical Center Hamburg-Eppendorf, Martinistraße 52, 20246 Hamburg, Germany

[3] Department of Radiology, Charité - Universitätsmedizin Berlin, corporate member of Freie Universität Berlin, Humboldt-Universität zu Berlin, and Berlin Institute of Health, Charitéplatz 1, 10117 Berlin, Germany

Supplementary Table S1. Computed tomography pulmonary angiography outcome per sector of patient care and priority group

		CT outcome	
		Negative	Positive
**Sector of patient care**			
	ER	732 (79.5%)	189 (20.5%)
	Inpatient	738 (83.9%)	142 (16.1%)
	ICU	170 (86.3%)	27 (13.7%)
	Reg. ward	568 (83.2%)	115 (16.8%)
	Outpatient	41 (85.4%)	7 (14.6%)
**Priority**			
	Not urgent	425 (84.8%)	76 (15.2%)
	Urgent	785 (80%)	196 (20%)
	Life-threatening	301 (82%)	66 (18%)
**All**		1511 (81.7%)	338 (18.3%)

Abbreviations: ER = emergency room; ICU = intensive care unit; reg. = regular.

Supplementary Table S2. Number of requests for CTPA by requester and distance from the requester to the respective CT scanner in meters.

**Sector of patient care**	**Distance to CT in meters**	**No. of requests per CT location**	
		ER	Department of radiology
**ER (3 locations)**			
	25	754	
	45	135	
	208		4
	238		28
**ICU (18 locations)**			
	39	3	
	57	2	
	98	28	
	101	23	
	128	19	
	130	21	
	159	18	
	166	20	
	216		1
	234		1
	236	19	
	241	8	
	249	24	
	275		3
	288		1
	307		1
	336		2
	343		1
	596	2	
**Regular ward (55 locations)**			
	55	1	
	65	55	
	66	56	
	74	27	
	83	32	
	84		4
	89		14
	95	6	
	107		1
	127		7
	190	7	
	211	45	
	221	39	

	227	35	
	230	86	
	233	25	
	241	50	
	249	104	
	260		7
	272	15	7
	275		3
	279		1
	280		1
	281		4
	288		9
	343		2
	432		2
	516	5	
	568	2	
	596	1	
	647	30	
**Outpatient (18 locations)**			
	49		3
	95	1	
	97	6	
	127		5
	172		2
	220	15	
	221	6	
	230	1	
	249	9	

Abbreviations: ER = emergency room; ICU = intensive care unit.

Supplementary Table S3. Triage categories per sector of patient care

	Priority			Total
**Sector**	Not urgent	Urgent	Life-threatening	
ER	201 (21.8%)	566 (61.5%)	154 (16.7%)	921
Inpatient	278 (31.6%)	393 (44.7%)	209 (23.8%)	880
ICU	79 (40.1%)	65 (33%)	53 (26.9%)	197
Reg. ward	199 (29.1%)	328 (48%)	156 (22.8%)	683
Outpatient	22 (45.8%)	22 (45.8%)	4 (8.3%)	48

Abbreviations: ER = emergency room; ICU = intensive care unit; reg. = regular.

Cases were assigned to three sectors of patient care (i.e., emergency room, inpatient, outpatient) based on the requesting department as specified in the digital request form in the radiological information system.

Supplementary Table S4. Multiple linear regression of ToR to ToS (ICU and regular ward specified).

	**ICU specified model**		
	exp^{β}	95% CI	P-value
Distance (per 100 m)	1.33	1.09 – 1.63	0.006
Sector of patient care			
ER vs. outpatient	3.46	1.16 – 10.36	0.026
ER vs. ICU	1.38	0.91 – 2.1	0.124
ER vs. reg. ward	2.13	1.74 – 2.62	<0.001
ICU vs. reg. ward	1.54	1 – 2.39	0.052
ICU vs. outpatient	2.5	0.78 – 7.99	0.122
Reg. ward vs. outpatient	1.62	0.54 – 4.89	0.391
Priority			
Not urgent vs. urgent	0.81	0.71 – 0.92	0.002
Not urgent vs. life-threatening	0.33	0.28 – 0.38	<0.001
Urgent vs. life-threatening	0.4	0.35 – 0.46	<0.001
CT requests at ToR (per 10 CTs)	1.05	1 – 1.1	0.034
CTs performed at ToR (per 10 CTs)	1.03	1 – 1.07	0.069
CT staff at ToR	1.12	1.08 – 1.15	<0.001
Occupancy at ToR	0.7	0.26 – 1.88	0.478
Interaction Sector of patient care & distance to CT			
ER (Reference)	1.33	1.09 – 1.63	0.006
Outpatient	0.76	0.42 – 1.37	0.36
ICU	0.81	0.6 – 1.08	0.154
Reg. ward	0.77	0.62 – 0.95	0.017

Abbreviations: CI = confidence interval; ER = emergency room; exp^{β} = exponentiated regression coefficient β to the base of *e*; ICU = intensive care unit; reg. = regular; ToR = time of request.

Supplementary Table S5. Average marginal effects of the distance from the requesting department to the CT scanner (per 100 meters) on the ToR to ToS for each sector of patient care (ICU and regular ward specified).

	**ICU specified model**		
	exp^{β}	95% CI	P-value
Sector of patient care			
ER	1.33	1.09 – 1.63	0.006
ICU	1.07	0.86 – 1.33	0.526
Reg. ward	1.02	0.95 – 1.1	0.545
Outpatient	1.01	0.58 – 1.75	0.972

Abbreviations: CI = confidence interval; ER = emergency room; exp^{β} = exponentiated regression coefficient β to the base of *e*; ICU = intensive care unit; reg. = regular.
